# Co-metabolism of thiocyanate and free cyanide by *Exiguobacterium acetylicum* and *Bacillus marisflavi* under alkaline conditions

**DOI:** 10.1007/s13205-016-0491-x

**Published:** 2016-08-18

**Authors:** Lukhanyo Mekuto, Oluwadara Oluwaseun Alegbeleye, Seteno Karabo Obed Ntwampe, Maxwell Mewa Ngongang, John Baptist Mudumbi, Enoch A. Akinpelu

**Affiliations:** 1 Bioresource Engineering Research Group, Department of Biotechnology, Cape Peninsula University of Technology, PO Box 652, Cape Town, 8000 South Africa; 2 Department of Microbiology, Agricultural Research Council, Private Bag X5026, Stellenbosch, 7599 South Africa

**Keywords:** *B. marisflavi*, Biodegradation, *E. acetylicum*, Free cyanide, Co-metabolism, Thiocyanate

## Abstract

The continuous discharge of cyanide-containing effluents to the environment has necessitated for the development of environmentally benign treatment processes that would result in complete detoxification of the cyanide-containing wastewaters, without producing additional environmental toxicants. Since biological detoxification of hazardous chemical compounds has been renowned for its robustness and environmental-friendliness, the ability of the *Exiguobacterium acetylicum* (GenBank accession number KT282229) and *Bacillus marisflavi* (GenBank accession number KR016603) to co-metabolise thiocyanate (SCN^−^) and free cyanide (CN^−^) under alkaline conditions was evaluated. *E. acetylicum* had an SCN^−^ degradation efficiency of 99.9 % from an initial SCN^−^ concentration of 150 mg SCN^−^/L, but the organism was unable to degrade CN^−^. Consequently, *B. marisflavi* had a CN^−^ degradation efficiency of 99 % from an initial concentration of 200 mg CN^−^/L. Similarly, the organism was unable to degrade SCN^−^; hence, this resulted in the evaluation of co-metabolism of SCN^−^ and CN^−^ by the two microbial species. Optimisation of operational conditions was evaluated using response surface methodology (RSM). A numeric optimisation technique was used to evaluate the optimisation of the input variables i.e. pH, temperature, SCN^−^ and CN^−^ concentrations. The optimum conditions were found to be as follows: pH 9.0, temperature 34 °C, 140 mg SCN^−^/L and 205 mg CN^−^/L under which complete SCN^−^ and CN^−^ degradation would be achieved over a 168-h period. Using the optimised data, co-metabolism of SCN^−^ and CN^−^ by both *E. acetylicum* and *B. marisflavi* was evaluated, achieving a combined degradation efficiency of ≥99.9 %. The high degradative capacity of these organisms has resulted in their supplementation on an active continuous biological degradation system that is treating both SCN^−^ and CN^−^.

**Electronic supplementary material:**

The online version of this article (doi:10.1007/s13205-016-0491-x) contains supplementary material, which is available to authorized users.

## Introduction

Cyanide is a naturally occurring compound which is produced by a variety of living organisms, such as fungi, plants, bacteria and algae (Luque-Almagro et al. 2005), and is also existent in the stratosphere and non-urban troposphere as a result of natural activities such as gases from volcanoes and burning of biomass. The contribution of these natural activities to cyanide contamination in the environment is insignificant as compared to anthropogenic activities. Anthropogenic activities contribute significantly to environmental deterioration because of the vast utilisation of cyanide compounds, which in turn generate extensive cyanide-containing wastewaters. Several industries such as the mineral processing, mining, electroplating and pharmaceutical industries contribute significantly to cyanide production worldwide. However, the mining industry accounts for 90 % cyanide utilisation, making it the largest cyanide utilising industry (Hu et al. 2015; Kitis et al. 2005; Kuyucak and Akcil 2013). Briefly, cyanide is used as a lixiviant for the recovery of gold, silver, and base metals (Cu, Ni, Zn, etc.) from gold-bearing ores in a process known as the cyanidation process (Habashi 1966, 1967). Gold-bearing ores are classified as sulphide ores, mainly due to the high presence of sulphides in such ores. During cyanidation, cyanide reacts with a variety of chemical constituents within the ore, thus forming a variety of cyanide complexes. The major chemical constituents found post-cyanidation are free cyanide (CN^−^) and thiocyanate (SCN^−^). SCN^−^ is formed as a result of CN^−^ reaction with sulphides and partially oxidised sulphur intermediates (Gould et al. 2012). The co-existence of CN^−^ and SCN^−^ in wastewaters is detrimental to both the environmental and living organisms, including humans.

Cyanide can be removed using chemical, photolytic, electrolytic, catalytic, ultrasonic and biological methods (Mudder et al. 2001; Sarla et al. 2004; Baxter and Cummings 2006). Biological methods have been proven to be environmentally friendly, robust and cost effective. Additionally, this method does not produce by/end-products that contribute to environmental destruction as compared to the aforementioned methods. In this process, CN^−^ and SCN^−^ are metabolised by microbial species to form ammonia, carbon dioxide and sulphates. The success of such a process lies in the ability of the microbial species to be applied in situ, under both aerobic and anaerobic conditions, in active and passive systems, and in suspended and attached growth systems (Akcil and Mudder 2003; Akcil et al. 2003). Furthermore, the microbial species can be manipulated to handle large influent flows and tolerate high CN^−^ and SCN^−^ concentrations, allowing for the uptake, catalysis, sorption and/or precipitation of CN^−^, SCN^−^, weak and strong acid dissociable cyanide complexes. However, this method has been hindered by the lack of thorough understanding of the individual microbial species, including the detoxification mechanism, which contributes to the successful degradation of CN^−^ and SCN^−^ (Huddy et al. 2015). The understanding of the intrinsic metabolic contributions of individual microbial species within a CN^−^ and SCN^−^ degrading consortia is paramount and, to achieve this, the metabolic activities of individual species need to be understood as these species employ different metabolic pathways for the degradation of CN^−^ and SCN^−^. These metabolic pathways include the hydrolytic, oxidative, reduction and substitution/transfer pathways (Gupta et al. 2010; Ebbs 2004; Dash et al. 2009). This information would add to the design of a high-strength microbial consortia that would ensure the maximum degradation of cyano-containing compounds. Some of the microbial species such as *Klebsiella oxytoca* are able to produce methane from CN^−^ degradation (Kao et al. 2003), thus demonstrating the economic value that can be achieved from biological degradation processes.

Individual microbial species have been explored for the degradation of CN^−^ and SCN^−^ and these include, *Klebsiella pneumoniae*, *Yersinia* sp., *Serratia marcescens* AQ07, *Aspergillus awamori*, *Burkholderia phytofirmans*, *Fusarium oxysporum*, *Thiobacillus thioparus*, *Trametes versicolor*, *Bacillus pumilus* and many other organisms (Chaudhari and Kodam 2009; Santos et al. 2013; Cabuk et al. 2006; Katayama et al. 1992; Meyers et al. 1991; Karamba et al. 2016; Vu et al. 2013; Akinpelu et al. 2016; Mpongwana et al. 2016). The CN^−^ and SCN^−^ degradative capacity of *Exiguobacterium acetylicum* and *Bacillus marisflavi* has never been reported before. Hence, this study focused on the co-metabolism of CN^−^ and SCN^−^ by *E. acetylicum* and *B. marisflavi* under alkaline conditions.

## Materials and methods

### Isolation and identification of the CN^−^ and SCN^−^ degrading bacteria

Bacterial species able to grow on media containing CN^−^ and SCN^−^ were isolated from the Diep River, Cape Town, South Africa. A culture-dependent technique was employed to isolate the organisms subsequent to serial dilutions of the original sample in saline solutions. This was followed by plating on nutrient agar containing 100 mg CN^−^/L and 100 mg SCN^−^/L, and incubation at 30 °C for 48 h, with an intention of isolating CN^−^ and SCN^−^-tolerant organisms. Two microbial organisms were selectively isolated, each from CN^−^ and SCN^−^-containing media.

The identification of the organism was performed using the 16S rDNA gene followed by polymerase chain reaction (PCR) in a thermal cycler (Mastercycler^®^ personal, Eppendorf AG, Germany). The DNA extraction and the subsequent amplification of the 16S rDNA gene were performed according to the method adapted from Mekuto et al. (2016). The PCR amplicons were purified and sequenced in a forward and reverse direction on the ABI PRISM^™^ 3500 analyser. The nucleotide sequences obtained were analysed using CLC main workbench 7 followed by a BLAST (Basic Local Alignment Search Tool) search provided by NCBI (National Centre for Biotechnology Information) (http://www.ncbi.nlm.nih.gov) and identified. The consensus sequences (supplementary Table S1) in FASTA format were deposited in the NCBI database and accession numbers were assigned as follows: KT282229 (*E. acetylicum*) and KR016603 (*B. marisflavi*).

### Seed culture preparation

Initially, the inoculum was prepared by inoculating single colonies of the isolates in Oxoid nutrient broth and agitated at 180 rpm in a shaking incubator set at 30 °C, for a period of 48 h. For subsequent experiments, the inoculum was prepared in minimal media (MM), without the presence of a nitrogen source. The MM contained (g/L): K_2_HPO_4_ (4.3), KH_2_PO_4_ (3.4), MgCl_2_.6H_2_O (0.4) and Acetate (0.1). For the optimisation studies, the cultures were grown at the conditions determined by Response Surface Methodology (see Table 2), for a period of 48 h prior to optimisation. The inoculum concentration was 10 % (v/v).

### Experimental plan

The initial degradation experiments were conducted using nutrient broth as media, to assess the biodegradative capacity of *E. acetylicum* and *B. marisflavi* at a CN^−^ and SCN^−^ concentrations of 200 mg CN^−^/L and 200 mg SCN^−^/L, respectively. The organisms were inoculated in MM that was supplemented with CN^−^ (as KCN) and SCN^−^ (as KSCN), in nutrient broth. *E. acetylicum* and *B. marisflavi* were both evaluated for the biodegradation of both CN^−^ and SCN^−^ separately. For the CN^−^ degradation studies, the pH of the media was at 9.5 since free cyanide is available in anionic state at this pH (Johnson, 2015). The SCN^−^ was conducted at a pH of 8.0. Due to the high degradation efficiencies that were obtained from MM, these media were utilised as growth media for the subsequent experiments. The effect of carbon source supplementation on the biodegradation process was assessed using glucose, fructose, acetate, starch and sucrose as sources of carbon. These experiments were run over a period of 72 h, at CN^−^ and SCN^−^ concentrations of 100 mg CN^−^/L and 100 mg SCN^−^/L, respectively.

### Response surface methodology: central composite design

A mathematical and statistical optimisation methodology, referred to as response surface methodology (RSM), was employed in this study to optimise the operational parameters that influence the CN^−^/SCN^−^ biodegradation process. This was achieved using the Design-Expert^®^ software (version 6.0.8, Stat-Ease Inc., Minneapolis, USA). The chosen operational parameters, i.e. pH, temperature, SCN^−^ and CN^−^ concentrations, were evaluated to determine the optimum operational conditions, which would result in complete degradation of SCN^−^ and CN^−^ in the same media. A 25-run experimental design was constructed using central composite design (CCD) at three levels: low (−2), medium (0) and high (+2) (see Table 1). The experimental design and their corresponding responses are tabulated in Table 2. All the experiments were conducted in 250 mL multiport airtight Erlenmeyer flasks and these flasks were used to minimise free cyanide volatilisation. The working volume was set at 100 mL using MM for growth and the experiment was conducted at 180 rpm at an inoculum concentration of 10 % (v/v) for a period of 168 h. The inoculum was prepared as described in the “Seed culture preparation” section, while the uninoculated flasks served as controls. The generated results from the biodegradation experiments served as a response (*Y*), as described in Eq. 1.

**Table 1:** Independent variables and levels used for central composite design

Variables	Code	Range and levels				
		+2	+1	0	+1	−2
pH	A	10.00	11	9.00	7.0	8.00
Temperature (°C)	B	37.0	40.5	33.50	26.5	30.0
Free cyanide (mg/L)	C	300	400	200	0.0	100
Thiocyanate (mg/L)	D	300	0.0	200	400	100

*α* = 2.0

**Table 2:** Central composite design using 3 variables and the corresponding response

Run	A	B	C	D	Degradation (%)
1	9.0	40.5	200	200	62
2	9.0	26.5	200	200	98
3	9.0	33.50	200	200	97
4	8.0	30.0	300	300	61
5	9.0	33.50	200	200	97
6	8.0	30.0	300	100	95
7	9.0	33.5	400	200	92
8	11.0	33.5	200	200	52
9	9.0	33.5	200	400	89
10	7.0	33.5	200	200	94
11	10.0	30.0	300	300	59
12	10.0	30.0	100	300	42
13	10.0	37.0	300	100	79
14	9.0	33.5	0.0	200	99
15	8.0	37.0	300	300	82
16	9.0	33.50	200	200	97
17	9.0	33.5	200	0.0	99
18	10.0	30.0	100	100	76
19	8.0	37.0	300	100	88
20	10.0	37.0	100	100	78
21	8.0	30.0	100	100	98
22	8.0	37.0	100	300	95
23	9.0	33.50	200	200	97
24	9.0	33.50	200	200	97
25	8.0	30.0	100	300	92
26	10.0	37.0	100	300	61
27	10.0	37.0	300	300	73
28	9.0	33.50	200	200	97
29	10.0	30.0	300	100	82
30	8.0	37.0	100	100	98

A, B and C represent the coded level of variables, while *α* represents the axial point with coded level of 2.0

1 $$Y = \beta_{0} + \sum {\beta_{i} X_{i} } + \sum {\beta_{ii} X_{i}^{2} } + \sum {\beta_{ij} X_{i} X_{j} + \varepsilon }$$

where $Y$ is the predicted response [degradation efficiency (%)], $\beta_{0}$ is the interception coefficient, $\beta_{i}$, $\beta_{ii}$ and $\beta_{ij}$ are the linear effect, quadratic and interaction coefficients, respectively. $X_{i}$ and $X_{j}$ are input variables that influence the degradation efficiency ($Y$), while $\varepsilon$ represents the random error.

Statistical analysis of the model was performed to evaluate the analysis of variance (ANOVA). The analysis included the overall model significance (*F* test), correlation coefficient *R*, and determination coefficient *R* ^2^, which measures the goodness of fit for the regression model. The response surface plots and contour plots were generated using Eq. 1.

The overall degradation efficiency for CN^−^ was calculated as proposed in Mekuto et al. (2016) with minor modifications. Instead of a 100× multiplication, a 50× multiplication was employed such that when combined with the 50× multiplication for SCN^−^ degradation efficiency (see Eq. 2), the overall degradation of the CN^−^ and SCN^−^ would account for 100 %.

2 $${\text{Biodegradation efficiency }}\left( \% \right) = \frac{{C_{\text{i}} - C_{\text{f}} }}{{C_{\text{i}} }} \times 50$$

where *C*_i_ and *C*_f_ represent the initial and final concentrations of free cyanide and thiocyanate

### Model validation

The accuracy of the predicted optimum conditions, which would ultimately result in over 99 % degradation efficiency as predicted by RSM, was assessed by evaluating the co-metabolism of SCN^−^ and CN^−^ by *E. acetylicum* and *B. marisflavi* at the optimised conditions. This was done to validate the predicted degradation efficiencies compared to the actual experimental data.

### Analytical methods

Merck cyanide (CN^−^) (09701), ammonium (NH_4_^+^) (00683), nitrate (14773) and sulphate (00617) test kits were used to quantify the concentration of free cyanide, ammonium, nitrate and sulphates using the Merck Spectroquant Nova 60 instrument. The principle behind the mechanism of detection by abovementioned test kits has already been elucidated elsewhere (Mekuto et al. 2016). Nitrites were determined according to Rider and Mellon (1946) while thiocyanate was detected using the ferric method (Katayama et al. 1992). The bacterial growth by pure cultures of the isolates was quantified using a Jenway 6715 UV/visible spectrophotometer at a wavelength of 600 nm.

## Results

### Isolation and identification of the CN^−^ and SCN^−^ degrading bacteria

**Fig. 1:**
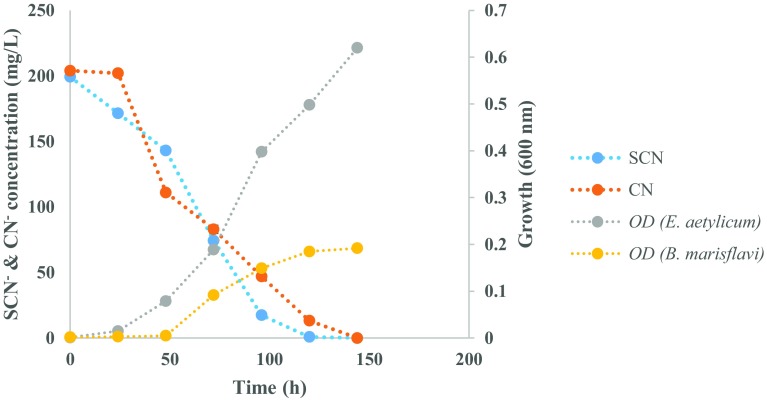
SCN^−^ and CN^−^ degradation profiles in nutrient broth media

**Fig. 2:**
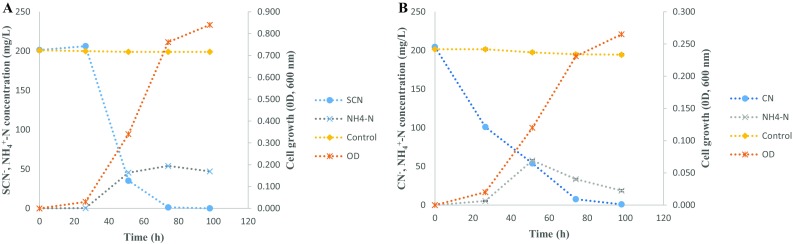
The SCN^−^ and CN^−^ degradation profiles by **a** *Exiguobacterium acetylicum* and **b** *Bacillus marisflavi* in minimal media

**Fig. 3:**
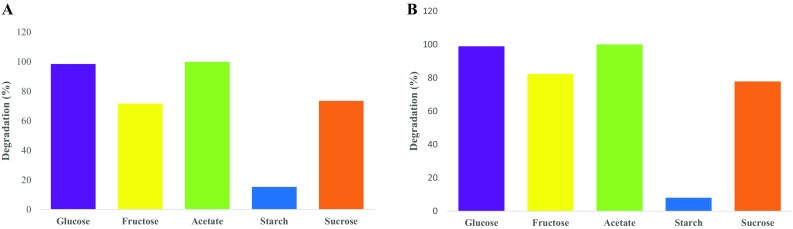
The effect of carbon source on SCN^−^ and CN^−^ degradation by, **a** *Exiguobacterium acetylicum* and **b** *Bacillus marisflavi*

A culture-dependent approach was utilised for the isolation of organisms that are able to tolerate and/or degrade CN^−^ and SCN^−^ separately, and two bacterial species were isolated and identified. *B. marisflavi* (GenBank accession number KR016603) degraded only CN^−^-containing media while *E. acetylicum* (GenBank accession number KT282229) degraded SCN^−^-containing media. However, these organisms were tolerant to the presence of these contaminants although they were incapable of degrading such contaminants. The ability of these organisms to degrade CN^−^ and SCN^−^ was evaluated where nutrient broth was used as the growth media. SCN^−^ degradation by *E. acetylicum* and its growth pattern in nutrient broth is shown in Fig. 1. The organism was able to achieve complete degradation of SCN^−^ from an initial concentration of 200 mg SCN^−^/L over a period of 144 h under slightly alkaline conditions, but was unable to degrade free cyanide (supplementary Fig. S1). On the other hand, CN^−^ degradation by *B. marisflavi* was evaluated under alkaline conditions, where the organism was able to degrade CN^−^ completely over a period of 144 h (Fig. 1). However, *B. marisflavi* was observed to be unable to degrade SCN^−^ (supplementary Fig. S1). Subsequent to the evaluation of these organisms’ ability to degrade CN^−^/SCN^−^ in nutrient broth, minimal media (MM) were evaluated as the potential growth media for the two organisms. These media were utilised as it contains minimal nutrient supplementation, thus ensuring minimal costs that are associated with nutrient supply. When MM was utilised as the growth media, *E. acetylicum* achieved complete degradation of SCN^−^ over a period of 98 h (Fig. 2a), while *B. marisflavi* achieved complete CN^−^ degradation over a 98-h period (Fig. 2b). A variety of carbon sources were evaluated to assess the best carbon source for the two organisms, and acetate was observed to be the preferred carbon source as it resulted in ≥99.9 % CN^−^ and 99.8 % SCN^−^ degradation efficiencies by *E. acetylicum* and *B. marisflavi*, respectively (Fig. 3a, b). Media supplemented with glucose achieved similar results to those achieved in media with acetate. The utilisation of glucose was forfeited due to the presence of keto groups in glucose. Keto groups have the ability to destabilize the triple bonds that hold the carbon and nitrogen in free cyanide, resulting in the formation of ammonia and carbon dioxide (Luque-Almagro et al. 2005). Hence, the reported biodegradation efficiency might be due to the action of the keto groups. Therefore, acetate was utilised as a preferred carbon source.

### Response surface methodology: central composite design (CCD)

The relationship amongst the independent variables and their optimum conditions were evaluated using CCD. Parameters such as pH (A), operating temperature (B), initial CN^−^ concentration (C) and initial SCN^−^ concentration (D) were selected as the important input variables that can affect cyanide and thiocyanate biodegradation. Through the CCD method, the combined impact of these parameters was studied from the generated 30 run experimentation (see Table 2). The model equation, which is a second-order polynomial equation for CN^−^ and SCN^−^ degradation efficiency, was obtained by performing regression analysis (Eq. 3).

3 $$Y = 97.00 - 10.13A - 0.96B - 1.46C - 6.21D - 7.16A^{2} - 5.41B^{2} - 1.53C^{2} - 1.91D^{2} + 0.94AB + 5.81AC - 1.94AD + 0.063BC + 4.06BD - 0.56CD$$

**Fig. 4:**
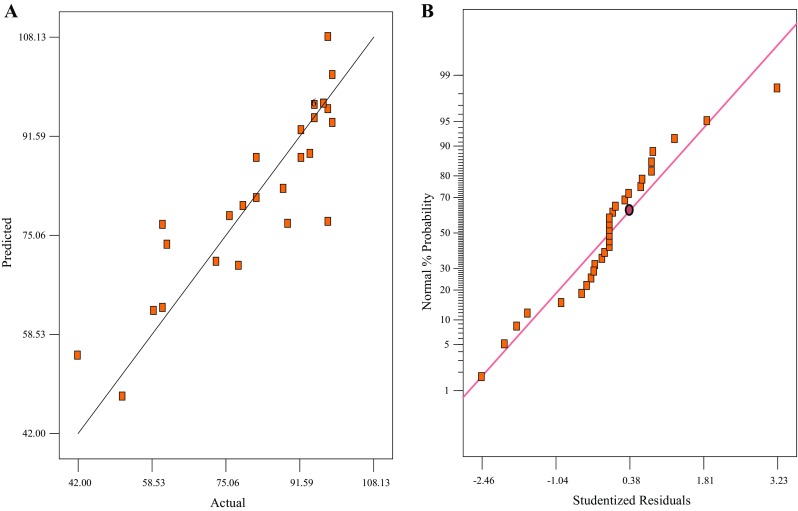
Graphical profiles representing, **a** the predicted and the actual values of CN^−^/SCN^−^ degradation efficiency, and **b** the normal probability of the studentised residuals

The *R* ^2^ of the regression equation was 0.90, indicating that the model was suitable for describing the co-metabolism of CN^−^ and SCN^−^ as a function of the selected factors (Fig. 4a). The regression model also revealed that the model explained 90 % of the experimental results. The high value of the adjusted determination coefficient indicated the significance of the model. The Fisher’s *F* test was used to assess the adequacy of the model and the results are shown in Table 3. The model *F* value of 4.55 implied that the model used was significant and the *P* values showed the significance of the coefficients, thus highlighting interactions between the variables. The adequate precision ratio of 8.536 obtained indicated an adequate signal while the lower value of the coefficient of variance (CV = 11.81) indicated better precision and reliability of the experiments. The confirmation of the adequacy and reliability of the generated model is paramount as it ensures sufficient representation of the actual test. Satisfactory normal probability plot of the residuals was achieved as the plots approximated along a straight line (Fig. 4b). The observed and predicted results based on the generated quadratic model (Eq. 2) are shown in Table 4.

**Table 3:** Analysis of variance (ANOVA) for the quadratic model

Source	Sum of squares	DF	Mean square	*F* value	Prob >*F*
Model	6288.55	14	449.18	4.55	0.0031
*A*	2460.38	1	2460.38	24.90	0.0002
*B*	22.04	1	22.04	0.22	0.6435
*C*	51.04	1	51.04	0.52	0.4834
*D*	925.04		925.04	9.36	0.0079
*A* ^*2*^	1404.67	1	1404.67	14.21	0.0019
*B* ^*2*^	801.67	1	801.67	8.11	0.0122
*C* ^*2*^	64.31	1	64.31	0.65	0.4324
*D* ^*2*^	99.67		99.67	1.01	0.3312
*AB*	14.06	1	14.06	0.14	0.7113
*AC*	540.56	1	540.56	5.47	0.0336
*AD*	60.06		60.06	0.61	0.4477
*BC*	0.062	1	0.062	0.000632	0.9803
*BD*	264.06		264.06	2.67	0.1229
*CD*	5.06		5.06	0.051	0.8240
Residual	1482.25	15	98.82	–	–
Lack of fit	1482.25	10	148.22	–	–

*R* ^2^ = 0.90, CV = 11.81, Adj. *R* ^2^ = 80.93

**Table 4:** Observed and predicted responses obtained using CCD

Run no.	Observed (mg/L)	Predicted (mg/L)
1	98.00	108.13
2	76.00	78.25
3	98.00	96.08
4	78.00	69.96
5	95.00	94.58
6	82.00	87.96
7	88.00	82.79
8	79.00	79.92
9	92.00	92.58
10	42.00	54.96
11	95.00	96.79
12	61.00	62.92
13	61.00	76.79
14	59.00	62.42
15	82.00	81.25
16	73.00	70.63
17	94.00	88.63
18	52.00	48.13
19	98.00	77.29
20	62.00	73.46
21	99.00	93.79
22	92.00	87.96
23	99.00	101.79
24	89.00	76.96
25	97.00	97.00
26	97.00	97.00
27	97.00	97.00
28	97.00	97.00
29	97.00	97.00
30	97.00	97.00

**Fig. 5:**
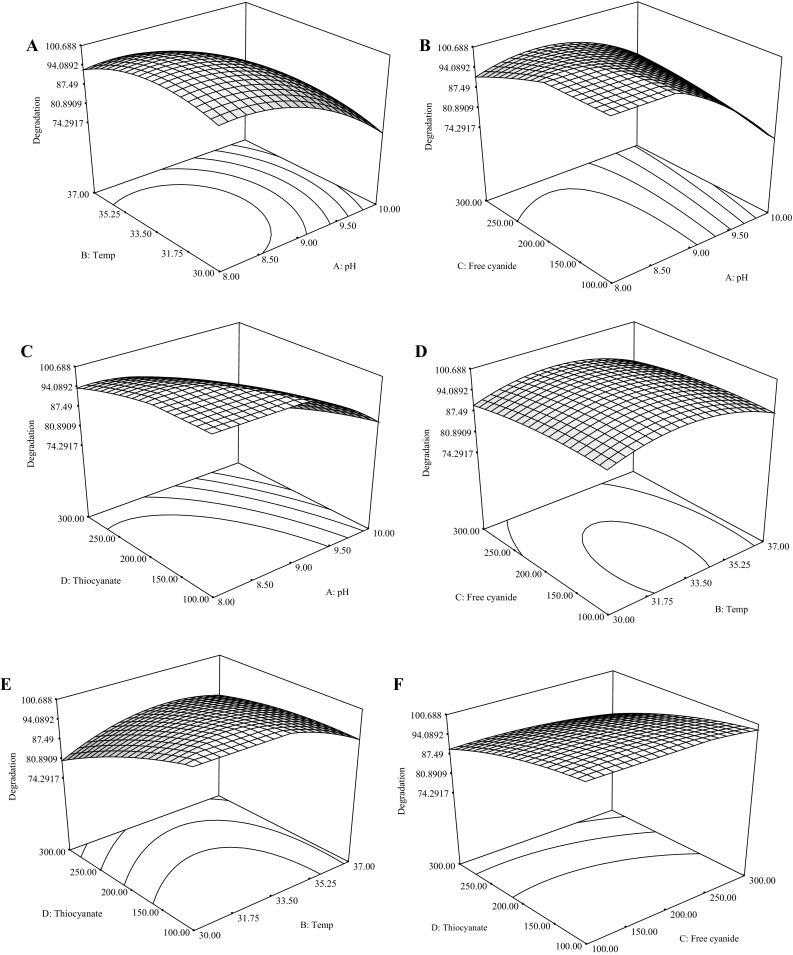
The response surface and contour plots of the interactions of, **a** temperature and pH, **b** Free cyanide and pH, **c** thiocyanate and pH, **d** Free cyanide and temperature, **e** thiocyanate and temperature and, **f** thiocyanate and free cyanide

The interaction between the tested variables was determined by plotting the response surface curves. 3D response surface and the 2D contour plots are the graphical demonstrations of the regression equation, and both are represented in Fig. 5a–f. The main goal of RSM is to obtain optimum conditions of the tested variables in such a way that the response is maximised. The resulting optimal responses for pH, temperature, CN^−^ and SCN^−^ concentrations were found to be 9.0, 34 °C, 140 mg SCN^−^/L and 205 mg CN^−^/L, respectively, with a desirability of 0.96, where an overall degradation efficiency of over 99 % can be achieved over an incubation period of 168 h.

**Fig. 6:**
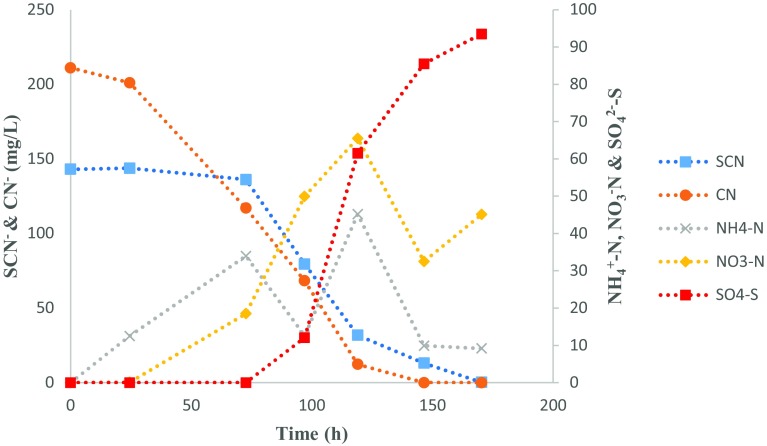
Model validation graphical profile on the co-metabolism of SCN^−^ and CN^−^

### Model validation

The accuracy of the predicted model was evaluated. Using the optimum conditions, the co-culture of *B. marisflavi* and *E. acetylicum* was assessed for the co-metabolism of CN^−^ and SCN^−^ in a batch system. The organisms completely degraded CN^−^ and SCN^−^ over a period of 170 h (Fig. 6). The degradation of these compounds was accompanied by the production of ammonium, nitrates and sulphates. There was an observed decrease in the concentrations of ammonium and nitrates after 120 h, suggesting the utilisation of these compounds by the microbial species, while the sulphates accumulated throughout the experimental run, producing the maximum sulphate concentration of 93.5 mg SO_4_^2−^-S/L. These results confirmed the accuracy and reliability of the generated model.

## Discussion

The present paper is the first report on the cyanide degrading capacity of *B. marisflavi* and thiocyanate biodegradation by *E. acetylicum*. Since *B. marisflavi* was unable to degrade SCN^−^ and the incapacity of *E. acetylicum* to degrade CN^−^, a co-metabolism study was undertaken to assess the effectiveness of co-culturing for the overall biodegradation of both CN^−^ and SCN^−^ in the same media. The co-cultures effectively degraded CN^−^ and SCN^−^ under alkaline conditions. Chaudhari and Kodam (2009) have shown that a co-culture of *Klebsiella pneumoniae* and *Ralstonia* sp. was able to degrade high thiocyanate concentrations, achieving degradation rates of 500 mg L^−1^ h^−1^. Consequently, 34 °C, pH 9.0, CN^−^ concentration of 140 mg CN^−^/L and SCN^−^ concentration of 205 mg SCN^−^/L were determined to be the appropriate physicochemical conditions for the maximum co-metabolism of CN^−^ and SCN^−^. Through RSM, *Bacillus* sp. CN-22 was observed to have optimum conditions at pH 10.3 and temperature of 31 °C, and CN-22 was able to tolerate up to 700 mg CN^−^/L. *Bacillus* species have been observed to be highly tolerant to high CN^−^ concentration in batch and continuous systems. Mekuto et al. (2015) assessed a consortia of *Bacillus* sp. in a continuous mode using a packed bed reactor under RSM-optimised conditions of: pH 9.88 and temperature of 33.8 °C. The authors observed a degradation efficiency of over 99 % when CN^−^ concentration was gradually increased from 100 to 500 mg CN^−^/L over a period of 80 days. *E. acetylicum* and *B. marisflavi* have been observed to be tolerant to CN^−^ and SCN^−^ concentrations of 300 mg CN^−^/L and 300 mg SCN^−^/L in the same media (see Table 2), making these organisms one of the few that tolerate the co-existence of CN^−^ and SCN^−^ at high concentrations. Furthermore, there are limited studies on SCN^−^ degradation under alkaline conditions. A symbiotic relationship was observed between the two organisms. This may be as a result of the close genetic relatedness of the species belonging to the *Exiguobacterium* and *Bacillus* genera (Farrow et al. 1992, 1994; Yumoto et al. 2004), thus resulting in the observed compatibility of the two organisms.

## Conclusion

This study focused on the co-metabolism of the free cyanide and thiocyanate by the isolated *B. marisflavi* and *E. acetylicum*. Analysis of the generated data revealed that the organisms successfully degraded both free cyanide and thiocyanate, achieving over 99 % degradation efficiencies. This demonstrated a symbiotic relationship between the two organisms. Using Response Surface Methodology, the optimum pH, temperature, free cyanide and thiocyanate concentrations were found to be 9.0, 34 °C, 140 mg CN^−^/L and 205 mg SCN^−^/L, respectively. Using these data, the generated model was validated through batch experiments, and the organisms completely degraded free cyanide and thiocyanate over 170 h, under alkaline conditions. This confirmed that the generated model is accurate and reliable. This is the first report on the co-metabolism of free cyanide and thiocyanate under alkaline conditions. Furthermore, this is the first report on thiocyanate and free cyanide by *E. acetylicum* and *B. marisflavi*. The information generated in this work will contribute to the construction of an effective microbial community that will ultimately contribute to the successful degradation of cyanide and thiocyanate wastewaters. As a result of the effectiveness of these organisms, they were supplemented in a continuous cyanide and thiocyanate degradation system that is currently ongoing, to aid in the successful destruction of these chemical compounds.

## Electronic supplementary material

Below is the link to the electronic supplementary material.

Supplementary material 1 (DOCX 77 kb)
