# Supplementary material for: Co-metabolism of thiocyanate and free cyanide by Exiguobacterium acetylicum and Bacillus marisflavi under alkaline conditions
Source: 3 Biotech. 2016 Aug 18;6(2):173. doi: 10.1007/s13205-016-0491-x (PMC4990519; doi:10.1007/s13205-016-0491-x)
Supplement: Supplementary file 1 — Supplementary material 1 (DOCX 77 kb) [file 13205_2016_491_MOESM1_ESM.docx]

**Supplementary data**

**Table S1: Consensus sequences of the isolated microorganisms**

| *Exiguobacterium acetylicum*  TCCTTRCGGTTACCTCACCGGCTTCGGGTGTTGCAAACTCTCGTGGTGTGACGGGCGGTG  TGTACAAGACCCGGGAACGTATTCACCGCAGTATGCTGACCTGCGATTACTAGCGATTCC  GACTTCATGCAGGCGAGTTGCAGCCTGCAATCCGAACTGGGAACGGCTTTATGGGATTGG  CTCCACCTCGCGGTCTCGCTGCCCTTTGTACCGTCCATTGTAGCACGTGTGTAGCCCAAC  TCATAAGGGGCATGATGATTTGACGTCATCCCCACCTTCCTCCGGTTTGTCACCGGCAGT  CTCCCTAGAGTGCCCAACTAAATGCTGGCAACTAAGGATAGGGGTTGCGCTCGTTGCGGG  ACTTAACCCAACATCTCACGACACGAGCTGACGACAACCATGCACCACCTGTCACCATTG  TCCCCGAAGGGAAAACTTGATCTCTCAAGCGGTCAATGGGATGTCAAGAGTTGGTAAGGT  TCTTCGCGTTGCTTCGAATTAAACCACATGCTCCACCGCTTGTGCGGGTCCCCGTCAATT  CCTTTGAGTTTCAGCCTTGCGGCCGTACTCYCCCAGGCGGAGTGCTTAATGCGTTAGCTT  CAGCACTGAGGGGCGGAAACCCCCCAACACCTAGCACTCATCGTTTACGGCGTGGACTAC  CAGGGTATCTAATCCTGTTTGCTCCCCACGCTTTCGCGCCTCAGCGTCAGTTACAGACCA  AAGAGTCGCCTTCGCCACTGGTGTTCCTCCACATCTCTACGCATTTCACCGCTACACGTG  GAATTCCACTCTTCTCTTCTGTACTCAAGCCTTCCAGTTTCCAATGGCCCTCCCCGGTTG  AGCSGGGGGCTTTCACATCAGACTTAAAAGGCCGCCTGCGCGCGCTTTACGCCCAATAAT  TCCGGACAACGCTTGCCACCTACGTATTACCGCGGCTGCTGGCACGTAGTTAGCCGTGGC  TTTCTCGTAAGGTACCGTCAAGGTACGAGCATTACCTCTCGTACGTGTTCTTCCCTTACA  ACAGAGTTTTACGATCCGAAAACCTTCATCACTCACGCGGCGTTGCTCCATCAGACTTTC  GTCCATTGTGGAAGATTCCCTACTGCTGCCTCCCGTAGGAGTCTGGGCCGTGTCTCAGTC  CCAGTGTGGCCGATCACCCTCTCAGGTCGGCTATGCATCGTCGCCTTGGTGGGCCGTTAC  CTCACCAACTAGCTAATGCACCGCAAGGCCATCTCAAGGTGACGCCGAAGCGCCTTTCAT  CAGCGGACCATGCGGTCCGTTGAACTATCCGGTATTAGCTCCGATTTCTCGGAGTTATCC  CAATCCTTGAGGCAGGTTCCTTACGTGTTACTCACCCGTCCGCCGCTCATTCCRCTGCCT  TCCCTCCGAAGAGTTCCGTCAGTTCCTGCGCTCGA |
| --- |
| *Bacillus marisflavi*  GAGCGGATCGATGGGAGCTTGCTCCCTGAGATCAGCGGCGGACGGGTGAGTAACACGTGGGTAACCTGCCTGTAAGACTGGGATAACTCCGGGAAACCGGGGCTAATACCGGATAACACCTACCCCCGCATGGGGGAAGGTTGAAAGGTGGCTTCGGCTATCACTTACAGATGGACCCGCGGCGCATTAGCTAGTTGGTGAGGTAATGGCTCACCAAGGCGACGATGCGTAGCCGACCTGAGAGGGTGATCGGCCACACTGGGACTGAGACACGGCCCAGACTCCTACGGGAGGCAGCAGTAGGGAATCTTCCGCAATGGACGAAAGTCTGACGGAGCAACGCCGCGTGAGTGAAGAAGGTTTTCGGATCGTAAAACTCTGTTGTTAGGGAAGAACAAGTGCCGTTCGAATAGGGCGGCGCCTTGACGGTACCTAACCAGAAAGCCACGGCTAACTACGTGCCAGCAGCCGCGGTAATACGTAGGTGGCAAGCGTTGTCCGGAATTATTGGGCGTAAAGCGCGCGCAGGTGGTTTCTTAAGTCTGATGTGAAAGCCCACGGCTCAACCGTGGAGGGTCATTGGAAACTGGGGAACTTGAGTGCAGAAGAGGAAAGTGGAATTCCAAGTGTAGCGGTGAAATGCGTAGATATTTGGAGGAACACCAGTGGCGAAGGCGACTTTCTGGTCTGTAACTGACACTGAGGCGCGAAAGCGTGGGGAGCAAACAGGATTAGATACCCTGGTAGTCCACGCCGTAAACGATGAGTGCTAAGTGTTAGAGGGTTTCCGCCCTTTAGTGCTGCAGCTAACGCATTAAGCACTCCGCCTGGGGAGTACGGTCGCAAGACTGAAACTCAAAGGAATTGACGGGGGCCCGCACAAGCGGTGGAGCATGTGGTTTAATTCGAAGCAACGCGAAGAACCTTACCAGGTCTTGACATCCTCTGACAACCCTAGAGATAGGGCTTTCCCCTTCGGGGGACAGAGTGACAGGTGGTGCATGGTTGTCGTCAGCTCGTGTCGTGAGATGTTGGGTTAAGTCCCGCAACGAGCGCAACCCTTGATCTTAGTTGCCAGCATTCAGTTGGGCACTCTAAGATGACTGCCGGTGACAAACCGGAGGAAGGTGGGGATGACGTCAAATCATCATGCCCCTTATGACCTGGGCTACACACGTGCTACAATGGACGGTACAAAGGGCTGCAAGACCGCGAGGTTTAGCCAATCCCATAAAACCGTTCTCAGTTCGGATTGTAGGCTGCAACTCGCCTACATGAAGCTGGAATCGCTAGTAATCGCGGATCAGCATGCCGCGGTGAATACGTTCCCGGGCCTTGTACACACCGCCCGTCACACCACGAGAGTTTGTAACACCCGAAGTCGGTGAGGTAACCTTT |

**Fig. S1**: Free cyanide and thiocyanate degradation profile by *Exiguobcterium acetylicum* and *Bacillus marisflavi*.
